# The lemon illusion: seeing curvature where there is none

**DOI:** 10.3389/fnhum.2015.00095

**Published:** 2015-02-23

**Authors:** Lars Strother, Kyle W. Killebrew, Gideon P. Caplovitz

**Affiliations:** Department of Psychology, University of NevadaReno, NV, USA

**Keywords:** shape perception, object recognition, curvature, concavity, convexity, discontinuity, visual illusion

## Abstract

Curvature is a highly informative visual cue for shape perception and object recognition. We introduce a novel illusion—the *Lemon Illusion*—in which subtle illusory curvature is perceived along contour regions that are devoid of physical curvature. We offer several perceptual demonstrations and observations that lead us to conclude that the Lemon Illusion is an instance of a more general illusory curvature phenomenon, one in which the presence of contour curvature discontinuities lead to the erroneous extension of perceived curvature. We propose that this erroneous extension of perceived curvature results from the interaction of neural mechanisms that operate on spatially local contour curvature signals with higher-tier mechanisms that serve to establish more global representations of object shape. Our observations suggest that the Lemon Illusion stems from discontinuous curvature transitions between rectilinear and curved contour segments. However, the presence of curvature discontinuities is not sufficient to produce the Lemon Illusion, and the minimal conditions necessary to elicit this subtle and insidious illusion are difficult to pin down.

## Introduction

**Figure 1:**
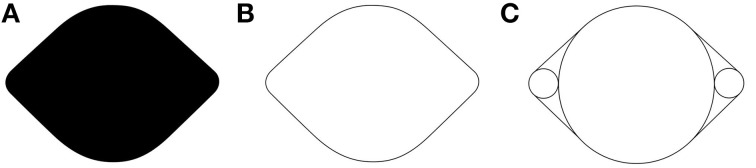
**The leftmost shape (A) resembles a lemon with subtle concavities between convex regions even though there are no physical concavities present**. Notice that the illusion occurs for **(A,B)** but not for **(C)**.

Visual illusions are fascinating and informative tools for vision science. In the spirit of this special issue concerning the relationship between illusions and neuroscience, we introduce a new visual illusion in which curvature is perceived where there is none. Figure 1A shows an example of a lemon-like shape with which the illusion was originally discovered, and from which we derived the name of the illusion—the *Lemon Illusion*. The Lemon Illusion refers to the subtle illusory concavities perceived in Figure 1A between each of its four convex regions.

Our fascination with the Lemon Illusion reflects a centuries-old interest in the role of curvature in visual perception and object recognition (e.g., Alhazen, 1030/1989). Of all potential cues to an object's shape, curvature is a particularly important type of visual information, in part because it provides cues about the three-dimensional surface structure of an object (Todd, 2004). Many theories of object recognition employ curved contours as shape primitives (Biederman, 1987; Ullman, 1989; Poggio and Edelman, 1990), and the human brain contains neurons that are tuned to specific degrees of curvature (Pasupathy and Connor, 2002). Curvature also plays an important role in the perceptual organization (Strother and Kubovy, 2006, 2012; Bertamini and Wagemans, 2013), and it may even play a low-level perceptual role in the way we feel about an object (Bar and Neta, 2006). Both the magnitude and sign of curvature are relevant to the perceived shape of an object. Curvature extrema exert a powerful influence on the salience of object parts (Hoffman and Singh, 1997). The perceptual effect of curvature magnitude is consistent with the idea that perceptual information is concentrated in regions of high curvature (Attneave, 1954), an idea that has been tested psychophysically and validated mathematically (Feldman and Singh, 2005; Lim and Leek, 2012; Singh and Feldman, 2012). The sign of curvature is also important to shape segmentation because positive (convex) curvature and negative (concave) curvature each signify different parts and part-boundaries within an object (Hoffman and Richards, 1984; Koenderink, 1984).

Here we offer several visual demonstrations and observations that highlight what we believe are the most important and informative conditions underlying the Lemon Illusion. We discuss our observations in relation to neural mechanisms that accomplish curve continuation, and we offer some insights into the relationship between these mechanisms and other aspects of shape perception and object recognition. We conclude that the illusory curvature in Figure 1A is not limited to perceived concavities in lemon-like shapes, but is instead an instance of a more general illusory curvature phenomenon, one that highlights the importance of curvature discontinuities in shape perception.

## Initial observations

The original observation of the Lemon Illusion entailed the perception of subtle concavities between the adjacent convexities of Figures 1A,B. Close inspection of Figures 1A,B will confirm that neither exhibits any physical concavities. Both Figures 1A,B were constructed by connecting the edges of circles using tangential line segments, shown explicitly in Figure 1C. Interestingly, the explicit representation of the circles prevents the illusion from occurring in Figure 1C.

*Observation 1:* The Lemon Illusion occurs for lemon-like silhouettes and outlines (Figures 1A,B). However, the illusion does not occur when circles and tangents comprising lemon-like figures are depicted explicitly (Figure 1C).

**Figure 2:**
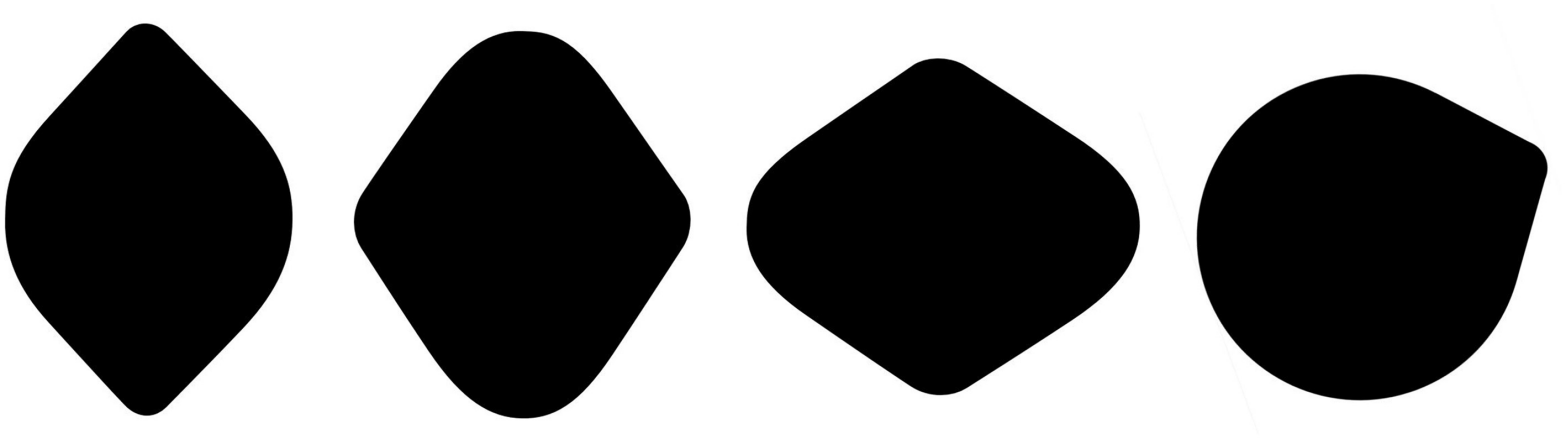
**The Lemon Illusion occurs for shapes that do not resemble lemons**. The rightmost silhouette shows that the illusion occurs for a shape with only two convexities.

*Observation 2:* The Lemon Illusion is not a familiarity effect—the illusion is not unique to lemon-like shapes or to shapes with only two convexities (Figure 2).

**Figure 3:**
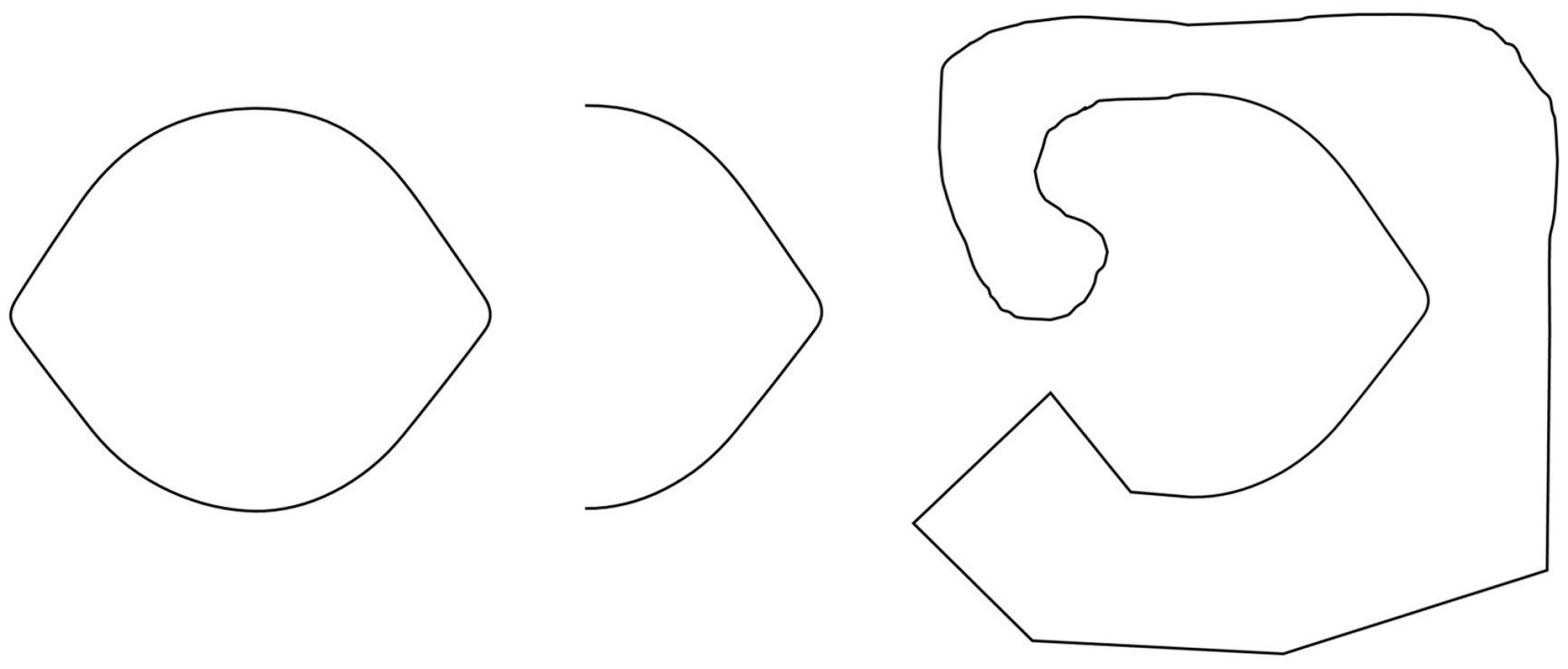
**The Lemon Illusion is not specific to closed contours; illusory curvature is also seen in the middle figure**. Figure-ground boundary assignment was reversed in the rightmost figure by joining the ends of a half-lemon (middle figure) with an arbitrary contour. The result is that a subtle convexity is perceived instead of a concavity.

*Observation 3:* The Lemon Illusion is not limited to closed contours or illusory concavities, and as such is not related to the sign of curvature (Figure 3).

**Figure 4:**
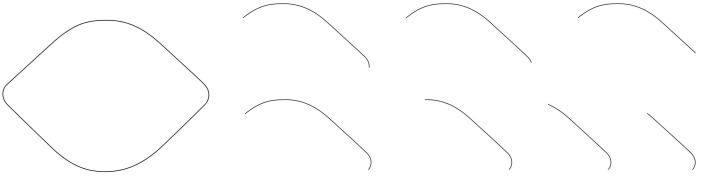
**The contours shown here were created by occluding portions of a lemon-like closed contour**. Although the Lemon Illusion persists as visible curvature is reduced, the illusion is either attenuated or absent for contours consisting of a single curve and rectilinear segment.

*Observation 4:* The Lemon Illusion is attenuated by reducing the amount of visible curvature in a contour consisting of two curves joined by a straight line, and possibly absent in the case of a single curve and a straight line (Figure 4).

**Figure 5:**
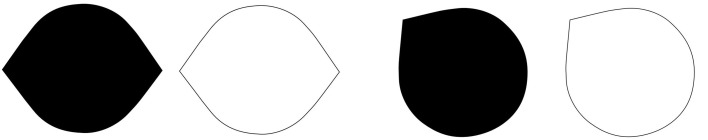
**The Lemon Illusion occurs for shapes with convexities that transition into a rectilinear contour segment, and then to a corner**.

*Observation 5:* The Lemon Illusion occurs for shapes with rectilinear contour regions bounded by a curve and a corner (Figure 5), and thus does not require two curved regions bounding a rectilinear contour segment (as one might predict based on Observation 4).

*Observation 5:* The Lemon Illusion is not limited to shapes with constant curvature (Figure 5).

Thus, far we have seen that, despite its name, the Lemon Illusion is not specific to lemon-like shapes. The Lemon Illusion occurs for silhouettes and contour lines, but for some reason it is abolished by the explicit representation of underlying geometry (Figure 1C). And although our original observation of the Lemon Illusion in Figure 1A led us to think that the illusion might be specific to illusory concavities, this hypothesis was disconfirmed (Figure 3), which means that the Lemon Illusion is not the result of potential perceptual anisotropies related to positive vs. negative curvature. Additionally, our observation that the combination of a single curve and a rectilinear contour segment fails to show the Lemon Illusion (Observation 5) led us to think that the illusion is unique to rectilinear contour segments bounded by two curves. Once again, however, we were wrong. Each of the shapes in Figure 5 consist of rectilinear contour segments bounded by a curve and a corner, and all four shapes elicit the Lemon Illusion. Finally, constant curvature does not appear to be a limiting factor (Observation 5); we return the importance of this shortly.

## The role of curvature discontinuities in shape perception

**Figure 6:**
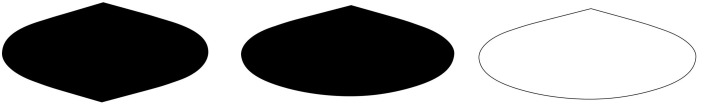
**The Lemon Illusion is seen in shapes with convex regions that do not have constant curvature**.

At this point it is worth noting that the instances of the Lemon Illusion demonstrated in Figure 1 through Figure 6 all elicit illusory curvature in the vicinity of a *curvature discontinuity* that occurs at the intersection of a curve and a straight contour segment. Contour curvature discontinuities are important cues for shape analysis, object recognition and motion perception (Attneave, 1954; Biederman, 1987; Feldman and Singh, 2005; Tse and Caplovitz, 2006). The visual system must make assumptions about the mapping between retinal images and the real world because two-dimensional (2D) retinal images are consistent with an indefinite number of three-dimensional (3D) real-world layouts. In the case of shape-analysis and object recognition, one important assumption is that an object is not being viewed from an “accidental” viewpoint—for instance, one in which a curved contour in the real world corresponds to a straight contour in a retinal image, which should only occur for a relatively limited number of viewpoints (i.e., it is unlikely to occur by chance). Thus, a curved contour in a retinal image will often be interpreted as a curved boundary in the real world.

*Observation 6:* The Lemon Illusion occurs in the vicinity of a curvature discontinuity.

**Figure 7:**
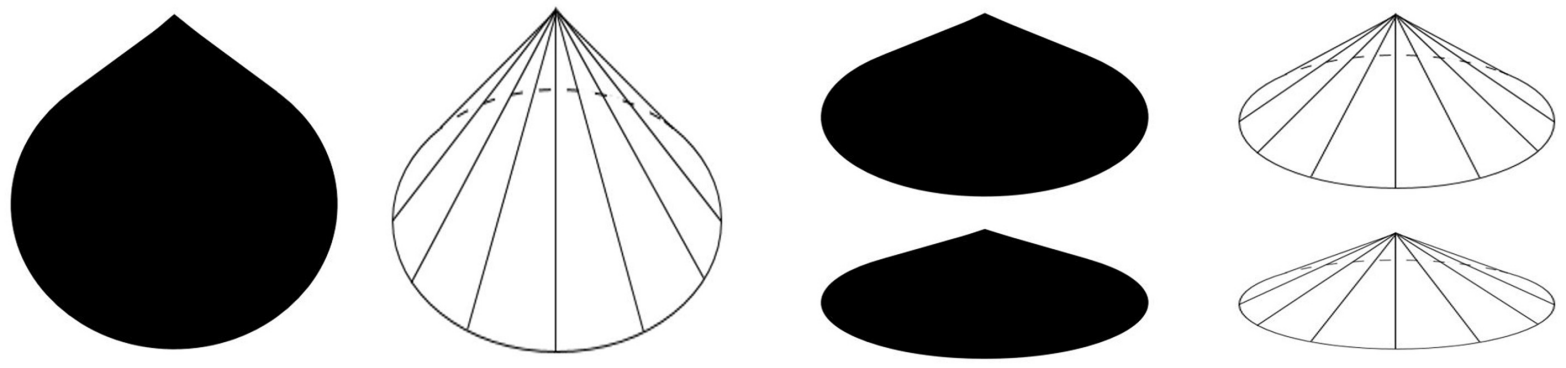
**The silhouettes shown here (and rightmost silhouette in Figure 5) can be interpreted as three-dimensional cones**. Curvature discontinuities indicate where the base of a cone becomes occluded (where dashed curves meet the line segments that join base and apex).

A consequence of the non-accidentality assumption is that curvature discontinuities are highly informative visual features that convey important information about the 3D form of an object (Kristjánsson and Tse, 2001). For instance, the leftmost silhouette in Figure 6 can be interpreted as a three-dimensional cone, with its vertex pointing up (Figure 7). When interpreted this way, the circular base must continue behind the surface of the cone (Figure 7, second from left, see dashed line). The point at which the base is occluded is the point at which there is a curvature discontinuity, which acts an indicator of a part-boundary between the base of the cone and the remainder of the cone formed by the locus of all straight line segments joining the apex to the perimeter of the base. Even if the leftmost silhouette in Figure 7 is not interpreted as a cone, it is easy to see a large round part that is perceptually distinct from a smaller tapered part. For either of these perceptual interpretations, at least two object parts are perceived (a “bottom part” and a “top part,”) the contour curvature discontinuity signaling the boundary between the two.

A similar many-to-one mapping of the real world onto the retinal image constrains the representation of object motion. Commonly referred to as the “aperture problem,” at the local level, an infinite number of 2D and 3D object trajectories can lead to identical patterns of activity in individual motion-sensitive neurons (Adelson and Movshon, 1982; Nakayama and Silverman, 1988a,b). This is particularly true for neurons whose receptive fields lie along the path of rectilinear or low-curvature regions of a moving object's contour. On the other hand, the 2D motions of contour and contour-curvature discontinuities are unambiguous and can be used by the visual system to overcome the aperture problem (Ullman, 1979; Caplovitz and Tse, 2006; Tse and Caplovitz, 2006). For the case of rotating objects, such discontinuities can disambiguate possible 3D form and motion representations (Sinha and Poggio, 1996) and provide a cue to the angular velocity of the object, a form of size-invariant motion constancy (Blair et al., 2014). Taken together, contour curvature and contour curvature discontinuities are key building blocks for a range of constructive perceptual processes. Not surprisingly there is evidence for neural populations selective for such discontinuities throughout visual cortex (Pack and Born, 2001; Pack et al., 2003, 2004; Caplovitz and Tse, 2007; Troncoso et al., 2007). It is thus, conceivable that the Lemon Illusion arises directly or epiphenomenally from constructive processes that rely on contour discontinuities, even discontinuities that occur along an unclosed contour.

## A possible neural basis of the lemon illusion

The Lemon Illusion presumably arises from the interaction of neural mechanisms involved in the visual processing of spatially local and global contour information. Physiological and neuroimaging research has shown that contour information is represented at the earliest stages of visual cortex. For example, neurons located as early as primary visual cortex (V1) have classical receptive fields specifically tuned to encode lines and rectilinear edges (Hubel and Wiesel, 1968). Similarly, there are neurons in V1 whose classical receptive field properties are well suited for representing the local curvature of a contour (Dobbins et al., 1987, 1989) and neurons in V2 specifically tuned to local contour curvature (Hegdé and Van Essen, 2000; Anzai et al., 2007). Neurons in later stages of visual cortex are able to process and represent more sophisticated contour characteristics across greater portions of the visual field. For example, neurons in visual area V4 can represent contour curvature and other contour features (Gallant et al., 1993, 1996; Pasupathy and Connor, 1999, 2001, 2002; Connor et al., 2007). These features can be integrated into entire object representations at still higher levels of ventral visual cortex (Ito et al., 1995; Missal et al., 1999; Kourtzi and Kanwisher, 2001; Grill-Spector and Malach, 2004; Strother et al., 2012).

Is the illusory curvature seen in the Lemon Illusion adequately explained by a low-level visual cortical mechanism? One hypothesis is that the illusory curvature perceived in the Lemon Illusion arises at an early stage of contour processing in retinotopic visual cortex (e.g., V1/V2). It is well known that the response of a visual cortical neuron to a stimulus presented in its classical receptive field can be modulated by the presence of distal stimuli. One source of extra-classical receptive field modulation can arise from the horizontal connections between neurons in early visual cortex (Rockland and Lund, 1983; Ts'o and Gilbert, 1988; Gilbert and Wiesel, 1989; McGuire et al., 1991), which can provide facilitatory input to neighboring neurons with similar orientation tuning (Singer and Gray, 1995), particularly those that are collinearly aligned (Kapadia et al., 2000; Sincich and Blasdel, 2001). These connections are thought to serve as the neural basis for so-called *association fields* (Field et al., 1993), which construct contours from spatially fragmented sources of contour information. It is possible that similar facilitatory mechanisms exist for neurons that represent local curvature (Poirier and Wilson, 2006), and that there be potentiation of neurons located along the curvature-continuation axis rather as well as the collinear axis. If this were the case, the population response to the rectilinear portion of contour would be biased toward the curvature-continuation axis. At higher stages of shape representation, this bias would need to be reconciled with other cues to the co-alignment of contour segments, ultimately leading to the erroneous perceptual extension of a curved contour segment—the Lemon Illusion.

It may be the case that neurons at mid-level stages of contour representation (i.e., V4) also contribute to the manifestation of the illusion. For example, neurons in V4 whose receptive fields are tuned to the processing of two convex projections separated by a concavity (Pasupathy and Connor, 2002) will be activated, albeit not-optimally, by stimuli in which the concavity is not present and instead consists of rectilinear contour. If there were a sufficiently greater number of neurons tuned to convex projections separated by concavities than rectilinear contours, then at the population-level, the output representation would be consistent with the presence of a concavity along the rectilinear portion of the contour. It has long been argued that neural mechanisms in higher-tier visual cortical areas represent hypotheses about low-level visual input, and in doing so, reinforce inferences (e.g., about shape) via feedback to lower level visual cortical mechanisms to facilitate efficient extraction and encoding of visual features (Engel et al., 2001; Murray et al., 2002; Cardin et al., 2011). It is therefore quite possible that The Lemon Illusion arises due to top-down feedback related to the representation of object shape manifest between shape-specific populations in V4 or even higher-level visual areas and early contour-continuation mechanisms in V1/V2. Still, there are some intriguing cases in which the Lemon Illusion does not occur (e.g., Figure 1C), and which may not be easily explained in terms of the neural mechanisms mentioned here, a challenge for future research.

## Special cases and future directions

Visual illusions have long played an important role in the vision sciences because they have the potential to foster insights into the neural mechanisms that underlie visual perception. Here we presented a novel visual illusion that highlights the prospective connections between perceptual phenomenology and the neural implementation of visual form analyses. We acknowledge up front that, while the Lemon Illusion is subtle, virtually everyone we have presented the illusion to reports the perception curvature where there is none. We even asked a group of students to copy a print-out version of the stimulus shown in Figure 1A. As can be seen in Figure S1, in almost all cases the hand-drawn copies included concavities although none were present in the image they were asked to copy.

**Figure 8:**
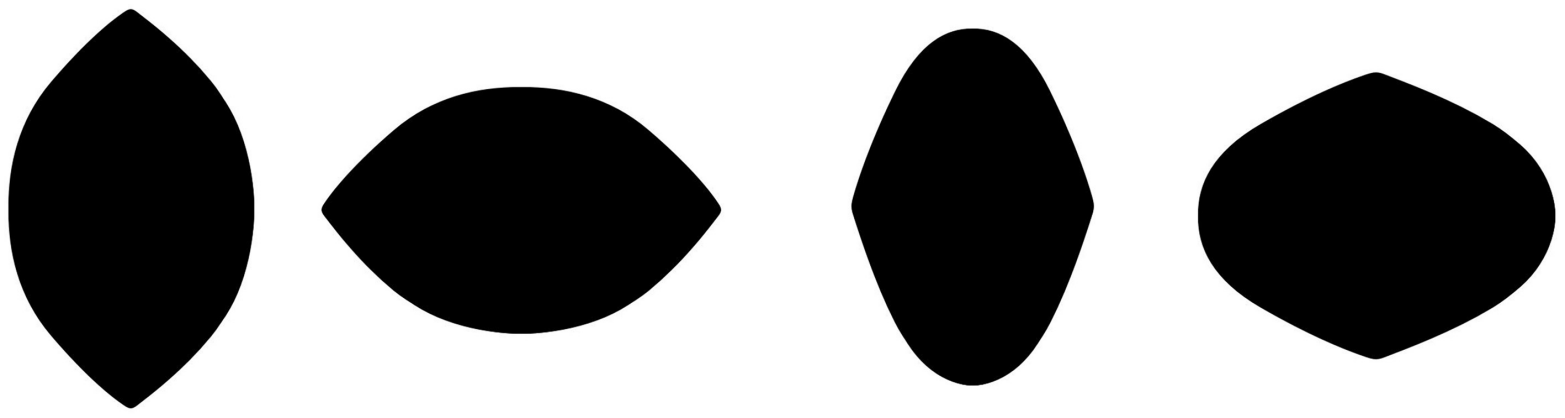
**The Lemon Illusion fails to occur when the transition between a decelerating (convex) curve and a rectilinear contour segment is sufficiently gradual**.

The primary goal of future research will be to identify the minimal conditions under which the Lemon Illusion occurs, and to develop experiments to test hypotheses about the neural mechanisms that underlie the illusion. This may be easier said than done. One promising approach could be to parametrically manipulate the degree of curvature deceleration into the rectilinear portion of a shape's bounding contour. As an example, notice that the Lemon Illusion fails to occur for the shapes in Figure 8, in which Bezier curves were used to reduce the abruptness of the discontinuity at the transition from convex curvature to the rectilinear portion of the contour. Even though there is still a rectilinear portion of the contour in each of the shapes in Figure 8, some of which resemble lemons, illusory concavities are not perceived—the transition from curved to rectilinear at the curvature discontinuity appears to be important in the Lemon Illusion.

**Figure 9:**
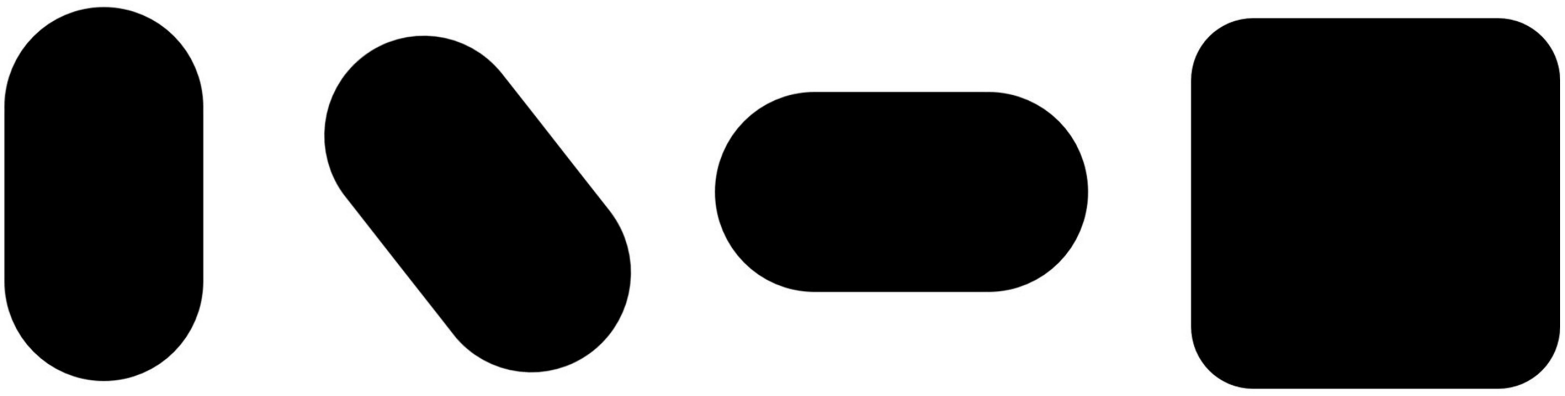
**The Lemon Illusion does not occur for these shapes, which have contour curvature discontinuities similar to those in other shapes that elicit the illusion (e.g., those in Figures 1, 2)**.

The observation that the Lemon Illusion can be abolished by modifying the change in the curvature of a contour as it approaches the zero curvature region means that, although constant curvature is not requisite for the Lemon Illusion to occur (as demonstrated earlier, in Figure 6), the rate at which curvature changes near a discontinuity may nevertheless be critical. This would be worth investigating further using a curve class that would allow for appropriate parametric manipulation. It is worth noting, however, that even if there is success with this method, some special cases will likely remain. For instance, we saw earlier that merely deleting the amount of visible curvature in two curved contour segments joined by a rectilinear contour segment appeared to weaken or abolish the Lemon Illusion (Figure 4). However, we also saw that introducing a corner in lieu of a curve is sufficient to restore the illusion (Figure 5). Similarly, even though we suspect that curvature discontinuities are of fundamental importance to the Lemon Illusion (Figures 6, 7), and to shape perception more generally, even the presence of curvature discontinuities does not necessarily suffice to induce the Lemon Illusion. Notice that none of the shapes in Figure 9 elicit the Lemon Illusion, even though each shape is comprised of highly similar combinations of rectilinear and curved contour segments used to create the shapes in Figures 1, 2.

In conclusion, the Lemon Illusion highlights the importance of contour curvature and curvature discontinuities in shape processing, both of which are essential to our ability to recognize 3D objects based on 2D shape information. While it is plausible that the illusion results from the interaction of early and higher-tier visual cortical mechanisms, clever experiments will be necessary to test specific hypotheses. The primary challenge for developing these experiments will be the identification of necessary geometric conditions under which the Lemon Illusion occurs.

### Conflict of interest statement

The authors declare that the research was conducted in the absence of any commercial or financial relationships that could be construed as a potential conflict of interest.
