# Supplementary material for: The lemon illusion: seeing curvature where there is none
Source: Front Hum Neurosci. 2015 Feb 23;9:95. doi: 10.3389/fnhum.2015.00095 (PMC4337333; doi:10.3389/fnhum.2015.00095)

1. **Supplementary Information**

**Figure S1.** We asked several naïve observers to draw a vertically oriented version of the lemon-like shape in Figure 4. Many observers drew concave contour regions, which suggests that they perceived concavities where there were none.


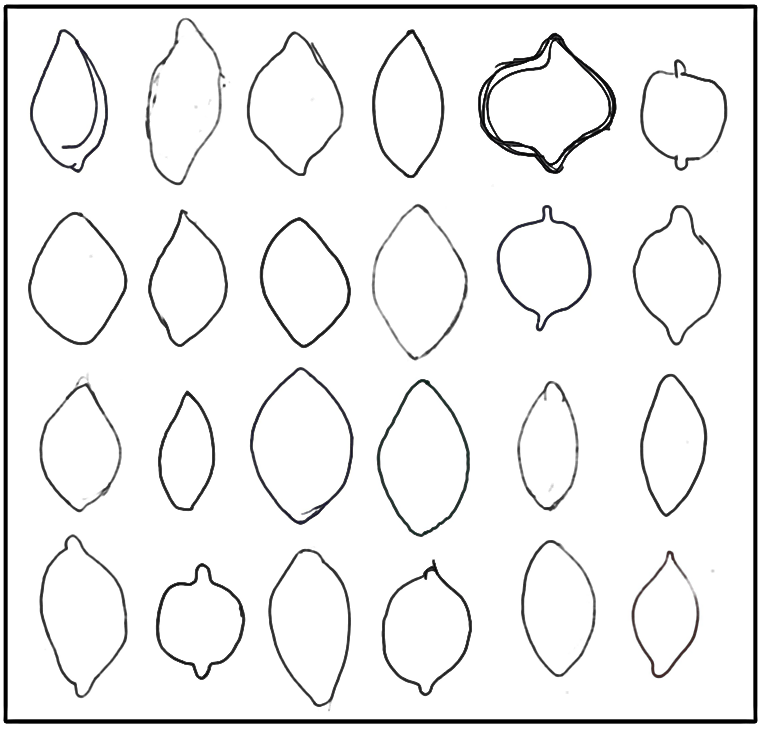

Supplement: Supplementary file 1 [file DataSheet1.DOCX]
